# Ecological opportunity and the evolution of habitat preferences in an arid-zone bird: implications for speciation in a climate-modified landscape

**DOI:** 10.1038/srep19613

**Published:** 2016-01-20

**Authors:** Janette A. Norman, Les Christidis

**Affiliations:** 1 National Marine Science Centre, Southern Cross University, Coffs Harbour, New South Wales, Australia; 2 Department of Genetics, University of Melbourne, Parkville, Victoria, Australia

## Abstract

Bioclimatic models are widely used to investigate the impacts of climate change on species distributions. Range shifts are expected to occur as species track their current climate niche yet the potential for exploitation of new ecological opportunities that may arise as ecosystems and communities remodel is rarely considered. Here we show that grasswrens of the *Amytornis textilis-modestus* complex responded to new ecological opportunities in Australia’s arid biome through shifts in habitat preference following the development of chenopod shrublands during the late Plio-Pleistocene. We find evidence of spatially explicit responses to climatically driven landscape changes including changes in niche width and patterns of population growth. Conservation of structural and functional aspects of the ancestral niche appear to have facilitated recent habitat shifts, while demographic responses to late Pleistocene climate change provide evidence for the greater resilience of populations inhabiting the recently evolved chenopod shrubland communities. Similar responses could occur under future climate change in species exposed to novel ecological conditions, or those already occupying spatially heterogeneous landscapes. Mechanistic models that consider structural and functional aspects of the niche along with regional hydro-dynamics may be better predictors of future climate responses in Australia’s arid biome than bioclimatic models alone.

Understanding how species will respond to climate change is of global concern. Current approaches emphasise the central role of the climatic niche and the impact of altered temperature and precipitation regimes on species distributions^1^. Geographical range shifts are expected as species track their contemporary climate niche^2^, with species inhabiting climate-sensitive landscapes considered at high risk of extinction^3^. Despite empirical evidence for rapid ecological and evolutionary adaptation^4^, comparatively few studies have investigated the potential for species to respond to new ecological opportunities that may arise under climate change, or their capacity to persist in climate-modified landscapes^5^.

Ecological opportunity occurs when a species enters a new environment following the extinction of competitors, the evolution of novel adaptations or the development of new ecological communities in disturbed or climate-modified landscapes^6^. Ecological opportunity can play a key role in structuring communities and promoting adaptive diversification^6^,^7^,^8^,^9^,^10^. New opportunities enable niche expansion through dispersal into novel environments^6^,^10^ which can lead to ecological divergence and speciation over evolutionary time scales. This occurs when habitats are spatially isolated or there is diversifying selection^7^,^8^,^9^. Ecological specialisation can also evolve as a response to changes in the extent of spatial and temporal environmental heterogeneity^11^. Finally, the degree of niche conservation is expected to influence the capacity of species to respond to new ecological opportunities; colonisations being rare or infrequent when conditions exceed the adaptive zone requirements and phenotypic variance of a species^10^.

Australia’s arid biome provides a unique opportunity to study how species have responded to new ecological opportunities arising from past climate change. This is a relatively young biome formed as the result of increasing aridity over the past six million years^12^,^13^ with major climate and landscape changes leading to the rapid evolution of distinctive arid-adapted vegetation communities^13^. Here we used grasswrens (Aves: Passeriformes) of the *Amytornis textilis*-*modestus* (ATM) species complex^14^ to test the hypothesis that shifts in habitat preference have occurred in response to the development of novel ecological opportunities in Australia’s arid biome. We applied an integrative approach in which Bayesian model testing was first used to evaluate statistical support for alternative evolutionary models that varied in terms of the number, sequence, and timing of habitat shifts required to explain the observed pattern of habitat preferences. We then evaluated the best-fit evolutionary model using time-calibrated molecular phylogenies of selected Australian Chenopodiaceae (saltbushes and bluebushes), in conjunction with paleobotanical and climate data, to determine if; (i) chenopod shrubland (CS) habitat preferences were ancestral or recently evolved, (ii) the extent to which ecological and evolutionary responses correlated with past climate fluctuations, and (iii) whether new ecological opportunities were associated with niche expansion (increased habitat diversity) or contraction (habitat specialisation). We also employed a population genetics approach to test for an association between habitat preference and demographic response to late Pleistocene climate change.

## Results

### Geographic distributions and habitat preferences of the ATM complex

**Figure 1:**
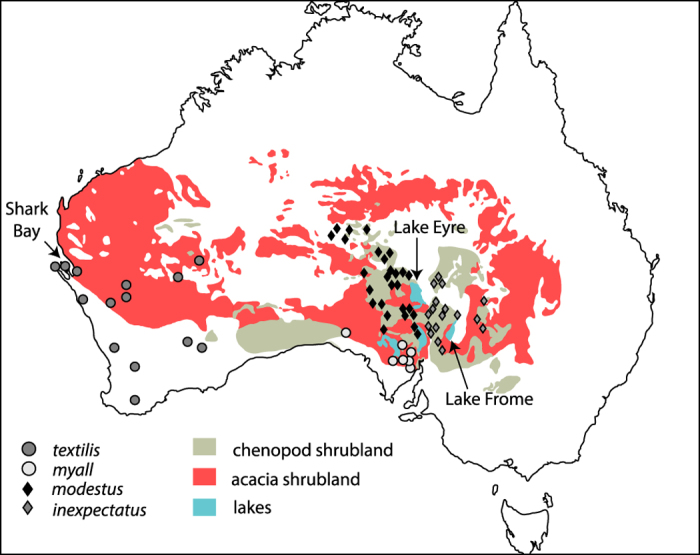
Geographical distribution and habitat associations in the ATM complex. The western subspecies of the Western Grasswren (*Amytornis textilis textilis,* 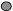) occurred in a wide range of shrubland habitats, most commonly acacia or eucalypt shrublands communities, but is now restricted to the far north-west of its range near Shark Bay. The eastern subspecies (*A. t. myall*, 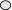) is most commonly associated with dense chenopod shrubland (CS) in the southernmost sector of the Lake Eyre Basin. The Thick-billed Grasswren (*A. modestus*) inhabits dense CS of central Australia with the subspecies *modestus* (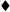) occupying drainages to the west of Lake Eyre, and *inexpectatus* (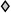) drainages to the east. Sample locations for the ATM complex ND2 sequences adapted from^20^. Vegetation map prepared by JN using Adobe Illustrator CS6 software and adapted from Journal of Arid Environments 75, S. R. Morton *et al.*, A fresh framework for the ecology of arid Australia, 313–329, 2011^59^, with permissions from Elsevier.

We compiled information on geographic distributions^15^,^16^ and habitat preferences^15^,^17^,^18^ from the literature. The ATM complex was formerly widespread across southern parts of the arid biome (Fig. 1) but habitat degradation due to pastoralism and competition from introduced pests (rabbits and goats) has resulted in the Western Grasswren (*Amytornis textilis*) being restricted to isolated populations at the eastern and western margins of its range. The western subspecies (*A. t. textilis*) occurred in a variety of shrubland habitats, most commonly acacia or eucalypt shrubland (AES) communities (Fig. 1) containing a dense understorey of low shrubs, but also dense thickets of lignum (*Muehlenbeckia*), saltbush and emu-bush (*Eremophila*) along drainage lines, and in patchily distributed tall dense chenopods. The flexible habitat associations of *textilis* contrast with those of the eastern subspecies (*A. t. myall*) which is largely restricted to CS dominated by black bluebush (*Maireana pyramidata*) in drainages of north-eastern Eyre Peninsula (Fig. 1). Similarly, the Thick-billed Grasswren (*A. modestus*) favours dense CS dominated by black bluebush and old man saltbush (*Atriplex nummularia*) with subspecies located in the Lake Eyre and Lake Frome drainages (*A. m. inexpectatus*), and the Finke River drainage (*A. m. modestus*) (Fig. 1). An extinct northern population of the subspecies *modestus* occurred in sandhill canegrass (*Zygochloa paradoxa*) habitat in the vicinity of the MacDonnell Ranges^17^. The ATM complex is largely absent from spinifex (*Triodia*) grasslands which are the dominant habitat association of most grasswren species^15^.

### Hypothetical models for the evolution of habitat preferences

**Figure 2:**
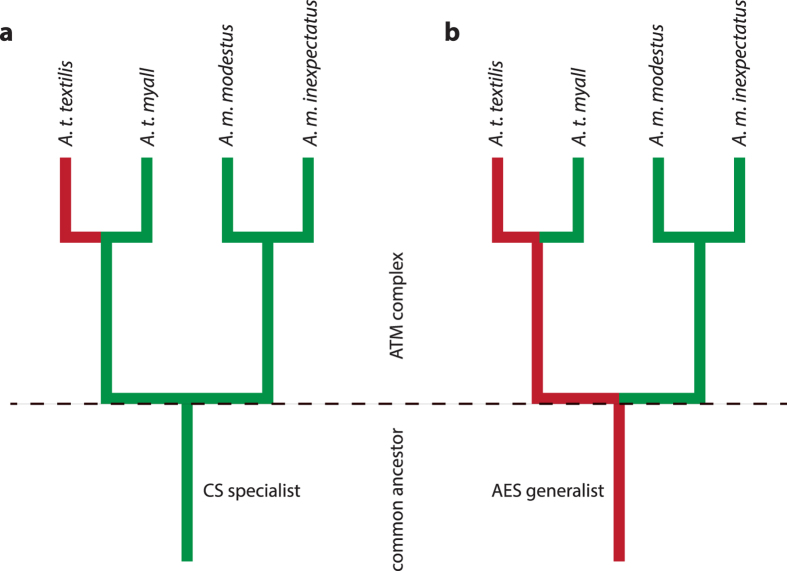
Alternative models for the evolution of habitat preferences in the ATM complex. Simplified phylogenetic framework based on analysis of mt ND2 sequences. The most parsimonious model (**a**) predicts that chenopod shrubland (CS) habitat preference (green) is the ancestral state with a recent transition to acacia-eucalypt shrubland (AES) habitat preference (red) in *textilis*. This model implies an increase in niche width (ecological release) in response to new ecological opportunity. The alternative model (**b**) predicts that AES habitat preference is the ancestral state with multiple transitions to CS under a model of constant migration; an early transition in *Amytornis modestus* with subsequent divergence of the subspecies *modestus* and *inexpectatus*, and a more recent transition in *myall*. This model implies a decrease in niche width and increased habitat specialisation in response to new ecological opportunity.

We constructed a phylogenetic framework for the ATM complex using published mitochondrial (mt) ND2 sequences^19^,^20^ (Supplementary Data 1 online) and used this to test alternative models for the evolution of habitat preferences (Fig. 2). The most parsimonious explanation is that CS was the ancestral habitat of the ATM complex with a single transition to AES in the subspecies *textilis* (Fig. 2a). This model predicts an increase in niche width associated with a recent expansion into AES. The alternative model, that AES were the ancestral habitat, allows for independent transitions to CS habitats during the evolutionary history of the complex (Fig. 2b). Assuming equal migration rates amongst ancestral (AES) and derived (CS) habitats this model predicts an early transition to CS in *A. modestus* and a more recent transition in *A. t. myall*. Alternatively, higher migration rates and/or weaker selection may have led to a slower rate of divergence in *myall* and an apparent temporal discrepancy in the timing of habitat shifts in the two lineages. Irrespective of the timing of divergence events, this model implies a decrease in niche width and increasing ecological specialisation associated with colonisation of CS.

An unconstrained model analysed in a Bayesian framework, and employing a reverse-jump hyper prior on transition rates, identified CS as the most likely ancestral habitat (57% likelihood) with limited support for AES (21%) (Supplementary Table S1 online). The model in which the most recent common ancestor (MRCA) of the ATM complex was constrained to CS also received greater support (Bayes Factor 1.7) than the model in which this node was constrained to AES (Bayes Factor 3.7). There was negligible support for *Triodia* (11%) as the ancestral habitat despite the former being the most common habitat association within the genus.

### Recent origin of CS communities

To test the hypothesis that CS was the ancestral habitat preference of the ATM complex (Fig. 2a) we constructed a temporal phylogeny for two lineages of the Australian Chenopodiaceae (*Atriplex* and Camphorosmeae) using published nuclear ITS sequences (Supplementary Data 1 online). These clades contain the species *A. nummularia* and *Maireana pyramidata*, dominant members of Australia’s CS communities and core habitat of the ATM complex in central Australia. We then used paleobotanical data^21^,^22^ to determine if the temporal evolutionary framework was consistent with the fossil history of Chenopodiaceae in Australia and establish a likely age for the formation of functional CS habitat in central Australia.

Given the wide range of age estimates reported for the origin of the Australian Chenopodiaceae^23^,^24^,^25^,^26^ (Supplementary Table S2 online), and uncertainties associated with the application of fossil calibrations^27^,^28^, we chose to date divergence times using an ITS rate calibration with a broad uniform prior. The prior encompassed, but was not constrained by, prior fossil calibrations. This returned a mean ITS rate of 0.0042 substitutions/site/lineage/million years (s/s/l/my) with similar 95% hyper posterior distributions (HPD) for both datasets (*Atriplex* 0.0014–0.0065, Camphorosmeae 0.0012–0.0066). The mean ITS rate was within the range reported previously^23^.

**Figure 3:**
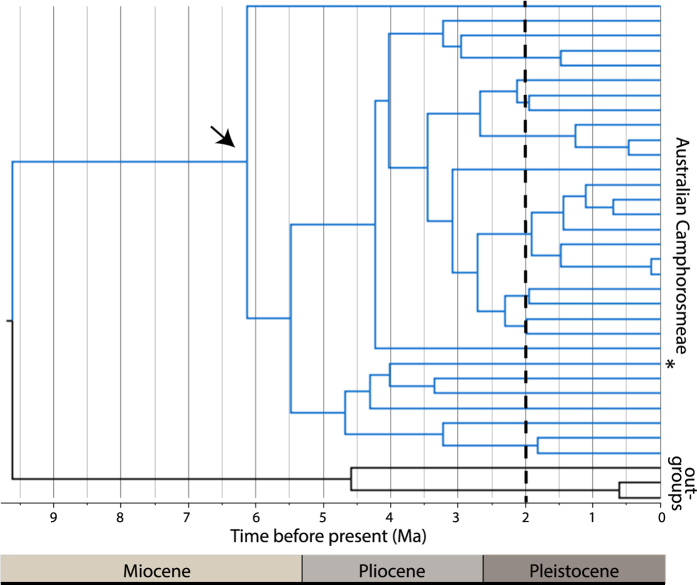
Temporally calibrated Bayesian maximum clade credibility trees based on ITS sequences for the Australian Camphorosmeae. The node corresponding to the onset of diversification of the Australian clade (blue branches) is shown (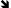) and commenced at 6.1 Ma before present. Bayesian posterior probability for this node was 0.95. Recent speciation events are clustered during the period 0.2–2 Ma as indicated by the dashed line and indicate the likely time period for the formation of contemporary CS communities. * denotes the species *Maireana pyramidata* a dominant species in the CS communities occupied by the ATM complex.

**Figure 4:**
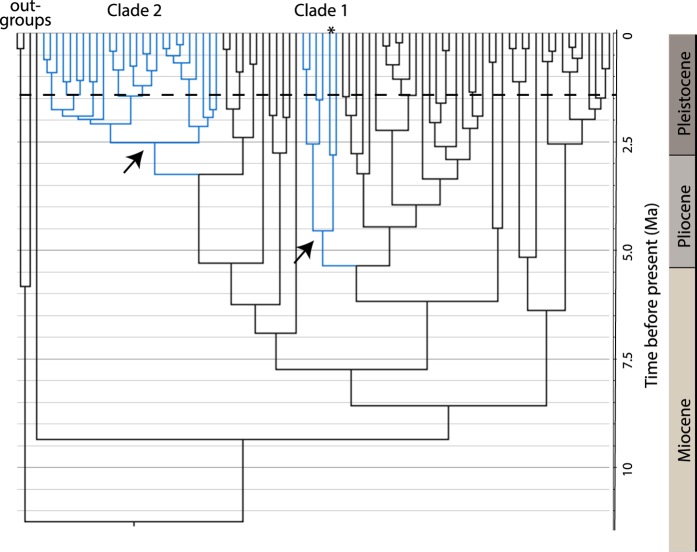
Temporally calibrated Bayesian maximum clade credibility trees based on ITS sequences for *Atriplex.* Australian clades are shown in blue. The nodes corresponding to the onset of diversification of the Australian clades are shown (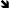) and are dated to 4.55 (Clade 1) and 2.52 Ma (Clade 2) before present. Bayesian posterior probabilities for these nodes were 0.95 and 0.98 respectively. * denotes the species *Atriplex nummularia* a dominant species in the CS communities occupied by the ATM complex.

The resulting temporal phylogenies (Figs 3 and 4) reconfirmed multiple origins for the Chenopodiaceae in Australia^23^ with independent colonisation events dated to the late Miocene (Camphorosmeae, ~9.6 Ma; Fig. 3), the Miocene-Pliocene boundary (*Atriplex* clade 1, ~5.45 Ma; Fig. 4), and the Pliocene (*Atriplex* clade 2, ~3.27 Ma, Fig. 4). As the formation of functional CS communities in central Australia must post-date the origin of these clades we used the onset of diversification (crown age), and the age of terminal lineages in each clade, to define plausible boundaries for the development of CS habitat. Progressive formation of CS is indicated, commencing in the late Miocene (~6.1 Ma) with rapid development during the Pliocene (~4.55 and 2.52 Ma) in association with the origin and radiation of *Atriplex*. However, formation of contemporary shrubland communities did not occur until the Pleistocene, as indicated by the clustering of recent speciation events at <2.0 Ma for Camphorosmeae (Fig. 3), and <1.0 Ma for *Atriplex* (Fig. 4). Incomplete taxon sampling precludes direct estimation of the origin of the two species that dominate CS habitat in central Australia. Nevertheless, the branches on which they lie are of relatively recent origin; 2.75 Ma for *A. nummularia* (Fig. 4) and 4.0 Ma for *M. pyramidata* (Fig. 3), with the phylogenetic position of the latter inferred from^30^. A previous study utilising ETS sequences, and calibrated using a root age of 16.3 Ma for the origin of the Australian Camphorosmeae^26^ (a date considerably older than the present study), returned an age of 3.0 Ma for *M. pyramidata*. Given the available evidence we conclude that both lineages are most likely of early Pleistocene origin.

Paleobotanical data^21^,^22^ support the proposed temporal framework. Earliest records of the Chenopodiaceae-Amaranthaceae alliance (chenopods) in Australia are from the Oligocene-Miocene but microfossils (pollen) from this period are rare and largely restricted to deposits formed in coastal and estuarine environments consistent with their arrival by littoral or Marine dispersal. Chenopods did not become widespread and abundant until the Pliocene with a dramatic increase in chenopod microfossils at multiple locations, including inland areas^21^. However, the persistence of rainforest trees in Pliocene deposits from Eyre Peninsula^22^ indicate that fully arid conditions had not yet formed in the eastern parts of the range currently occupied by the ATM complex. The combined data support the mid-Pliocene (~3.5–4.0 Ma) as a likely age for the initial formation of functional CS communities in Australia with contemporary habitats formed during the early Pleistocene (~2 Ma). Thus, CS represents a new ecological opportunity in Australia’s arid biome that was readily colonised by grasswrens of the ATM complex.

### Divergence times in the ATM complex and ancestral habitat preference

We next tested for temporal congruence between the estimated age for the formation of CS communities (~2–3.5 Ma) and the age span of the common ancestor of the ATM complex. We employed Bayesian coalescent analysis of the mt ND2 sequences to estimate (i) the time to common ancestry (T_MRCA_) for lineages in the ATM complex (coalescence of ND2 sequences of *Amytornis textilis* and *A. modestus*) and (ii) the age of the root node (divergence of the ATM complex from its sister lineages; *A. housei, A. goyderi, A. purnelli* and *A. ballarae*). The interval between them represents the age span of the common ancestor of the ATM complex. We also estimated T_MRCA_ for lineages within each species.

**Figure 5:**
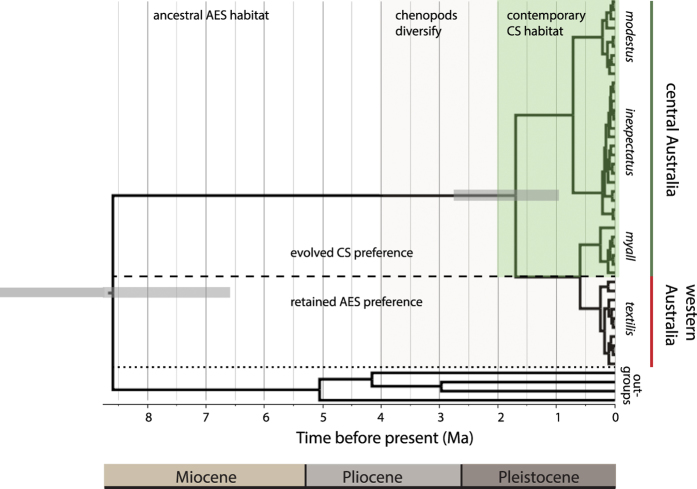
Time-calibrated phylogeny for the ATM complex and formation of chenopod shrubland habitats in central Australia. The tree was generated using a Bayesian coalescent analysis of 41 unique mt ND2 sequences detailed in the Supplementary Data. The common ancestor of extant lineages in the ATM complex was estimated to have occurred during the interval 8.6–1.7 Ma with 95% confidence intervals (grey bars) estimated using an avian ND2 rate calibration^51^. Chenopods diversified and increased in abundance during the Pliocene (light green shading) becoming widespread and abundant across inland Australia. The formation of contemporary CS habitat in central Australia (dark green shading) most likely occurred during the Pleistocene, and is associated with a shift in habitat preferences from AES to CS in *modestus*, *inexpectatus* and *myall*. The ancestral AES habitat preference is retained in *textilis* from western Australia. Outgroups occur in *Triodia* grasslands and sandhill canegrass habitats.

The analysis returned a mean age of 8.6 Ma (95% HPD 6.6–11.1 Ma; Fig. 5) for the divergence of the ATM complex from its sister lineage under a relaxed clock model. A mean T_MRCA_ of 1.7 Ma (95% HPD 1.0–2.4 Ma) was obtained for coalescence of *A. textilis* and *A. modestus* under a relaxed clock model (with outgroups), and 2.0 Ma under a strict clock model with outgroups excluded (95% HPD 1.4–2.7 Ma). T_MRCA_ for the four subspecies are dated to the middle Pleistocene with similar estimates for the relaxed and strict clock models; 0.63–0.64 Ma for the coalescence of *textilis* and *myall* (95% HPD 0.36–0.94 relaxed, 0.38–0.94 strict) and 0.72–0.79 Ma for the coalescence of *modestus* and *inexpectatus* (95% HPD 0.39–1.06 relaxed, 0.48–1.13 strict).

The root age of 8.6 Ma substantially predates the origin of functional CS communities (~2–3.5 Ma) and the origin of both *Atriplex* clades (~3.27 and ~5.45 Ma), indicating that CS were not the ancestral habitat of the ATM complex between 8.6 and 3.5 Ma. We reject the best-fit evolutionary model from the Bayesian model-testing procedure (Fig. 2a) and conclude that CS habitat preferences have evolved from populations inhabiting AES. The failure of independent (molecular) data to support the best-fit evolutionary model highlights the potential limitations of the comparative method in evolutionary studies.

Identification of AES as the ancestral habitat is consistent with an older evolutionary history for the endemic acacias and eucalypts in Australia, which originated over 25 Ma and diversified extensively in response to increasing aridity during the late Miocene and early Pliocene^29^,^30^,^31^. Paleobotanical data also indicate that central Australia was dominated by schlerophyll woodland and shrubland during this period but lacked the extensive grassland ecosystems that dominate the arid and semi-arid biome today^21^. Preliminary molecular data also support a recent origin for Australia’s arid grasslands with diversification of *Triodia* dated to 6.6 Ma (Supplementary Data 2 online). This post-dates the origin of the ATM complex and provides additional support for AES, or a transitional shrubland community, as the ancestral habitat preference. Finally, contemporary AES habitat associations in the ATM complex^15^ include three acacia species that occur along watercourses and floodplains (Dead Finish *Acacia tetragonophylla* and Limestone wattle *A. sclerosperma*) or the fringes of salt lakes in central Australia (Umbrella Bush *A. ligulata*). Occupation of similar landscape elements and the spatial proximity of CS and AES habitats would have facilitated the rapid colonisation of newly evolved CS communities by the ATM complex.

Our combined analyses demonstrate that the ATM complex responded to the development of CS as a new ecological opportunity through an initial increase in niche width. Subsequent habitat specialisation (niche contraction) occurred in populations inhabiting CS communities in central Australia and is associated with divergence of *A. modestus* and *A. t. myall*. While differences in the timing of shifts to CS are indicated, this disparity could result from higher levels of gene flow or diffuse selection limiting the rate at which *myall* has diverged^6^,^7^.

### Climatic influences on diversification and the evolution of new ecological opportunities

**Figure 6:**
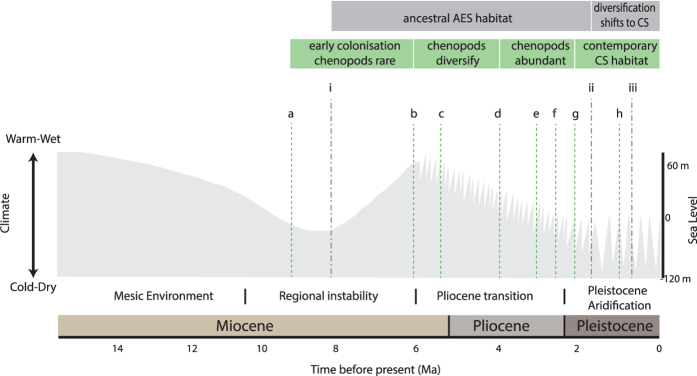
Correlations between paleoclimate change, the evolution of chenopod shrubland habitat, and the timing of shifts in habitat preference in the ATM complex. Climate profile for Australia adapted from^12^. The timing of events associated with the origin and diversification of the chenopods *Atriplex* and Camphorosmeae (a–e) were determined from Bayesian analysis of ITS sequences, and the fossil record of the Chenopodiaceae-Amaranthaceae alliance in Australia. Four phases in the evolution of chenopod shrubland (CS) habitats were identified: an initial phase marked by the origin of the Camphorosmeae (a) followed by a period of evolutionary stasis as climate returned to the warm-wet conditions typical of the Miocene; a second phase associated with the onset of diversification in Camphorosmeae (b) and colonisation by *Atriplex* clade 1 (c) as Pliocene aridification commenced; a third phase in which a dramatic increase in the abundance of chenopod fossils and the diversification of *Atriplex* clade 1(d) occurred, followed by the origin (e) and diversification (f) of *Atriplex* clade 2; a final phase associated with recent speciation events in Camphorosmeae (g) and *Atriplex* (h), indicating the formation of contemporary CS habitats, occurred during the Pleistocene as glacial climate oscillations intensified. The timing of diversification events in the ATM complex (i-iii) were determined from Bayesian analysis of mt ND2 sequences. The ancestor of the ATM complex arose during the Miocene (i) prior to the evolution of CS habitats. A shift from ancestral acacia-eucalypt shrubland (AES) habitat preference to CS is associated with the divergence of *A. textilis* and *A. modestus* (ii) during the early Pleistocene, with the divergence of subspecies (iii) occurring in the late Pleistocene. Figure prepared by JN using Adobe Illustrator CS6 software with the climate profile adapted from Molecular Ecology 17, M. Byrne *et al*., Birth of a biome: insights into the assembly and maintenance of the Australian arid zone biota, 4398-4417, 2008, with permission from John Wiley and Sons.

To determine if new ecological opportunities have developed in response to Plio-Pleistocene climate change we tested for temporal congruence between major climate events, the origin and diversification of chenopods, and speciation in the ATM complex in the arid biome (Fig. 6). The paleoclimate profile for Australia was adapted from the literature^12^ and temporal phylogenies based on our analyses of ITS and mtND2 sequences. The resulting alignment identified four major phases underlying the evolution of CS habitat preferences. The first corresponds to a period of climatic instability in the late Miocene from ~6–9 Ma which is associated with the initial development of aridity in central Australia, the origin of the Camphorosmeae, and the early evolution of the ATM complex in ancestral AES shrubland habitats. The second and third phases occurred during the Pliocene and are associated with the onset of cyclic climate variability and widespread aridification in Australia. The origin and diversification of *Atriplex* clades 1 and 2 occurred during this period, with chenopods becoming widespread and abundant during the mid-Pliocene. The final phase corresponds to increased climatic variability during the Pleistocene and is associated with speciation in the ATM complex and the formation of contemporary CS communities.

### Habitat preferences and niche width influence demographic responses to late Pleistocene climate fluctuations

Our analyses demonstrate that ecological opportunity has led to increased habitat specialisation and a decrease in niche width in subspecies of the ATM complex inhabiting CS. Niche specialisation is predicted to occur in environments that are relatively stable in both space and time^11^. According to this model, populations of the ATM complex in CS occupy a more stable environment than those in AES. This should lead to differences in the long-term demographic responses of populations to common environmental perturbations with measurable impacts on levels and patterns of genetic diversity^32^ and effective population size^33^,^34^. To test this hypothesis we used population genetic approaches to determine if genetic signatures of late Pleistocene demographic stability or expansion were evident in subspecies of the ATM complex inhabiting CS.

**Figure 7:**
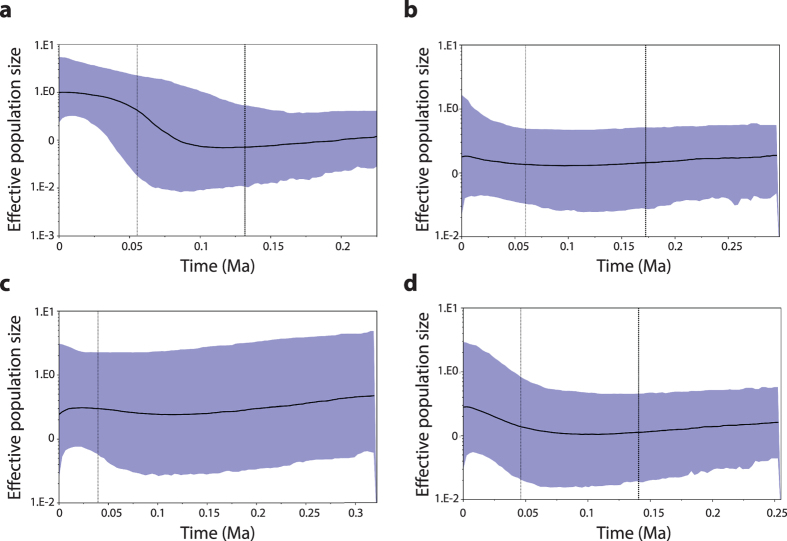
Bayesian skyline plots of late Pleistocene demographic responses in subspecies of the ATM complex. Median estimate of log effective population size with 95% confidence intervals (shaded) are shown for *inexpectatus* (**a**), *modestus* (**b**), *textilis* (**c**) and *myall* (**d**). A late Pleistocene population decline is evident in *textilis* from AES commencing at the last glacial maximum approximately 25,000 ka. In subspecies inhabiting CS modest population growth (*myall* and *modestus*) or population expansion (*inexpectatus*) are indicated.

Bayesian skyline plots (Fig. 7) indicate continuous late Pleistocene population growth in the three populations inhabiting CS with a period of strong demographic expansion observed for *inexpectatus* between ~120 ka and ~40 ka. A similar pattern of modest population expansion was also evident in *textilis* prior to ~100 ka with a significant reduction in effective population size occurring since the last glacial maximum (LGM) ~25,000 years ago. Our analysis supports the predictions of ecological theory with populations inhabiting CS buffered to a greater extent against late Pleistocene climate instability.

## Discussion

We identify chenopod shrubland (CS) habitats of Australia’s arid biome as a new ecological opportunity that developed in response to increased aridification during the Plio-Pleistocene. Consistent with theoretical expectations, the emergence of CS as a new ecological opportunity precedes (or is closely correlated with) lineage-splitting events^8^ in the ATM complex confirming ecological opportunity as a driver of speciation. Ecological opportunity has played a key role in structuring ecological communities in Australia’s arid biome by providing critical habitat for grasswrens of the ATM complex^15^,^18^ and numerous other birds, mammals and reptiles in the arid biome^16^,^35^,^36^. It is associated with increased ecological specialisation in the ATM complex and has contributed to the demographic resilience of populations inhabiting CS habitats.

Conservation of structural and functional aspects of the ancestral niche^10^ may have facilitated colonisation of CS habitats by the ATM complex. Structurally, AES and CS are classified as low dense shrubland which appears critical for the provision of necessary resources and a suitable microclimate. Rapid utilisation of new ecological opportunities can also occur in species with broad adaptive zones^10^, or as a response to spatial heterogeneity when there is competition for resources^37^. The diversity of habitats utilised by the ATM complex suggest that their adaptive zone requirements are able to be met by a variety of structurally similar vegetation communities. This includes sandhill canegrass, a shrub-like plant from the Poaceae (grasses)^38^ and preferred habitat of the extinct northern population of *modestus*. The observed spatio-temporal patterns are consistent with an evolutionary bet-hedging strategy^39^ in which the ATM complex retains the ability to shift habitat preferences in the face of fluctuating environmental conditions. Such dynamics can lead to instability in ecological and evolutionary outcomes when environments change^8^. As a result, the periodic breakdown of species boundaries and frequent alterations to community composition may be salient features of evolution in the arid biome.

The combination of broad adaptive zone requirements and conservation of structural and functional aspects of the ancestral niche suggest that multiple diffuse selection pressures^7^, rather than strong adaptive divergence, underlie shifts in habitat preferences in the ATM complex. Plausible targets for selection include release from interspecific competition and predation pressures, access to novel resources, and a reduction in thermal and osmotic stress. Shrubs provide significant amelioration of local environmental conditions in desert ecosystems and contribute to the diversity of ecologically important microclimates^40^. They modify soil and air temperature, humidity, wind velocity, solar radiation and soil evaporation^41^. Even minor differences in these parameters can be significant for ground-dwelling birds^42^ and a source of diversifying selection. Interspecific competition is also implicated with central Australia the centre of diversity for grasswrens with geographic replacements typically occupying distinctive vegetation communities^15^.

Deterministic processes (selection) may also underlie the distinct demographic responses of populations of the ATM complex from CS habitats. In particular, the physiognomy of saltbush and bluebush may facilitate the resilience that buffers these populations from short-term climate extremes and long-term climate fluctuations. *Atriplex nummularia* and *Maireana pyramidata* are large, deep-rooted shrubs that are both drought- and salt-tolerant. They are able to access water resources not available to other species including saline groundwater^43^,^44^. These adaptations are associated with an increased tolerance of chenopod to extended periods of water deficit^45^ and higher temperatures^46^ than many other arid-zone plants. The development of CS in the Lake Eyre Basin, an endorheic (closed) drainage system, may also contribute to the long-term stability of this ecosystem. The basin receives episodic flows derived from two remote synoptic systems, the northern monsoon and the temperate westerlies. These episodically flood the rivers and creeks that drain into the Lake Eyre Basin, recharging the shallow saline groundwater systems^47^, and potentially decoupling plant and soil water balances from local precipitation events. Future climate predictions of reduced precipitation across southern Australia^48^ may have little direct impact on the persistence of CS in the Lake Eyre Basin. Modification of groundwater levels and recharge rates associated with changes in monsoonal weather patterns are likely to be more important for the persistence of CS which may function as ecological and/or evolutionary refugia for the survival of the numerous arid-adapted species that inhabit central Australia’s CS communities.

Our efforts to discern the ecological and evolutionary processes underlying recent shifts in habitat preferences in the ATM complex provide new insights into the role of ecological opportunity in promoting species persistence and diversification in Australia’s arid biome. Despite a recent origin, chenopod shrubland has become one of the dominant vegetation communities of the arid biome and core habitat for the ATM complex along with numerous other birds, mammals and reptiles. The capacity of the ATM complex to respond to this new ecological opportunity appears to be determined by multiple interacting factors including the adaptive zone requirements of the species, the spatial proximity of the habitats and conservation of structural and functional similarities between them, including provision of a suitable microclimate through amelioration of local environmental conditions. As vegetation communities are patchily distributed within Australia’s arid biome continued habitat degradation will limit the capacity for the endemic fauna to respond to future climate change by increasing spatial isolation between structurally and functionally similar elements and decreasing habitat complexity leading to dispersal constraints and increased competition for limited resources. To reliably predict the persistence of Australia’s arid-adapted fauna under future climate change will require the development of mechanistic models that account for at least some of these processes, including the potential for the unique hydrodynamic properties of the Lake Eyre Basin to effectively decouple species responses from local climate events.

## Methods

### Phylogenetic framework for the ATM complex

We sourced mt ND2 sequences from the NCBI database to construct a phylogenetic framework for studying the evolution of habitat preferences in the ATM complex. The dataset comprised 74 sequences (Supplementary Data 1 online) obtained from both extant and extinct populations sampled from across the geographic range of these species^20^, which included 41 unique ATM haplotypes. Representative sequences from four taxa that comprise the sister lineage to this complex^19^ were included as outgroups. The best-fit model of sequence evolution for the 41 unique ATM haplotypes was determined using Kakusan 4^49^ with the preferred model identified as HKY+Γ. The best-fit model for the same dataset with outgroups included was TN93+Γ.

Both datasets were tested for departures from neutral expectations of a strict molecular clock. Comparisons of the strict and uncorrelated lognormal relaxed (UCLR) clock models were performed in BEAST 1.8^50^ using the stepping stone path sampling procedure. Topological constraints were enforced for the ATM complex according to relationships inferred in previously published phylogenies^19^,^20^. Two putative *myall* sequences that clustered with *textilis* in a previous analysis^20^ were included in the latter. A coalescent constant population size tree prior was applied and the initial MCMC run for 10 million generations with the first 10% discarded as burn-in. The path-sampling procedure was then used to derive marginal log likelihood values using the posterior distribution of the MCMC. The sampling procedure contained 100 steps and 10 million generations with a 10% burn-in. Each analysis was run twice to ensure convergence and maximum clade credibility trees computed from the data. The analysis with outgroups returned a smaller marginal log likelihood for the UCLR clock model (strict -3355, UCLR -3352), with slightly elevated ND2 rates (range 0.0141–0.0150 s/s/l/My) observed for the outgroup taxa *A. goyderi* and *A. purnelli* (Supplementary Fig. S2 online). Similar marginal log likelihoods were obtained for the models when outgroups were excluded (strict -2217, UCLR -2218) confirming a uniform evolutionary rate for ND2 sequences within the ATM complex.

### T_MRCA_, population size effects and demographic expansion

Estimates of coalescence times to the most recent common ancestor (T_MRCA_) for the ATM complex were estimated in BEAST 1.8 using a dataset comprising all 74 ND2 sequences. Previous efforts to date divergence times in *Amytornis*^19^ and the ATM complex^20^ employed incorrect calibrations of 0.02 and 0.029, respectively. These values are the divergence rates between lineages, and not the per lineage substitution rate, resulting in node ages being substantially underestimated. In the present study we used the avian ND2 rate of 0.0145 substitutions/site/lineage/million years (s/s/l/MY)^51^.

Analyses employed the TN93+Γ model of sequence evolution, a constant population size tree prior, and a strict molecular clock with a normal prior on clock rate with mean and standard deviation of 0.0145 and 0.0007 s/s/l/my, respectively. The MCMC was run for 10 million generations and the initial 10% discarded as burn-in. Each analysis was performed twice to ensure convergence of independent runs, and ESS values >200 for all parameters. The analysis was repeated with outgroups excluded, and a HKY+Γ model of sequence evolution applied to the data, to investigate how model and data parameters affected T_MRCA_ estimates.

To test for differences in historical demographic processes we constructed Bayesian skyline plots^52^ for each subspecies using BEAST 1.8. A strict clock model with a HKY+Γ substitution model was employed with the same normal prior on clock rate and MCMC parameters as above. The Bayesian Skyline coalescent tree prior was implemented as a piecewise-stepwise model with group size of five.

### Habitat preference and ancestral state reconstructions

Ancestral state reconstructions for habitat preference were performed in a Bayesian framework^53^ using BayesTraits V2.0 with details of habitat preferences for the ATM complex and outgroups sourced from the literature^15^. As descriptions of habitat preferences may be confounded by misidentification of species in early historical accounts we also relied on the reviews of^17^,^18^ to clarify details of species distributions, taxonomy and habitats. For simplicity, habitat preference was treated as a multistate character with four states based on the dominant vegetation communities occupied by each species or subspecies; (a) chenopod shrubland (CS; *modestus*, *inexpectatus* and *myall*), (b) acacia-eucalypt shrubland communities (AES; *textilis*), (c) spinifex (*Triodia*) grassland (outgroups *A*. *purnelli*, *A*. *ballarae* and *A*. *housei*) and (d) sandhill canegrass (*Zygochloa paradoxa*) (outgroup *A*. *goyderi*).

For ancestral state reconstructions we employed a simplified phylogenetic hypothesis in which each taxon was represented by a single branch (with lengths equal to the mean branch length for that clade determined in our phylogenetic analysis). While this approach does not take phylogenetic uncertainty into account, Bayesian support for monophyly of species and subspecies in the ATM complex is high^20^. To evaluate the potential effect of over-parameterisation of the models we first conducted a maximum likelihood analysis comparing a null model, in which all transition rates were fixed to be equal (1-parameter), with an unconstrained model in which all rate transitions were independently estimated (12-parameters). The Likelihood Ratio Test identified the simpler model as a better fit to the data. We subsequently employed a reversible-jump MCMC procedure that allows sampling of the various possible models of evolution in proportion to their posterior probabilities. This approach obviates the need for empirical optimisation of restrictions on rate transitions.

To evaluate alternative models for the evolution of habitat preferences we fixed the MRCA of the ATM complex to CS or AES (Fig. 2). We employed a gamma distributed hyperprior (0 1, 0 1) to ensure that the range of rate transitions obtained from the unconstrained (12-parameter) analysis were contained within, but not determined by, the prior range. The MCMC was run for 5,050,000 generations with the initial 50,000 discarded as burn-in. Sampling was conducted every 1,000 iterations to produce 5,000 data-points for the posterior distribution and the model run twice to ensure the harmonic means had converged to similar values. Mixing of the MCMC was monitored to ensure adequate exploration of parameter space, with an acceptance rate of approximately 30% achieved for all parameters. The models were evaluated using Bayes Factor comparisons calculated as twice the difference in log-harmonic mean of the complex (unconstrained) model and the simpler constraint models.

### Chenopod ITS phylogeny

Previous molecular phylogenetic studies of the Chenopodiaceae^23^,^24^,^25^,^26^ have returned a range of possible dates for the origin and diversification of *Atriplex* and the Camphorosmeae in Australia (Supplementary Table S2 online). Variability in these estimates likely results from differences in the phylogenetic resolution of the genomic regions examined, problems arising from the alignment of highly divergent sequences across hypervariable regions, and variation in the application of fossil node constraints. Uncertainty concerning the appropriateness of fossil calibrations employed in these studies must also be considered. In particular, the use of *Parvangula randeckensis* (a *Chenopodium*-like seed dated to 23.3–16 Ma) to calibrate the crown age of the *Chenopodium* clade referred to as Chenopodieae 1^23^, has not been justified; *Chenopodium* being polyphyletic and constituting at least four distinct clades^23^. Additionally, the use of relatively deep nodes as fossil calibrations may result in large errors in the estimation of node ages for recent lineages (the focus of this study), due to the effects of time-dependent molecular evolution^27^. Phylogenetic placement and the temporal accuracy of fossil calibration represent the most significant contributions to imprecision in the estimation of node ages^28^.

To minimise these potential sources of error and variability we compiled 609–618 bps of nuclear ITS sequences (*ITS1*-5.8s rRNA-*ITS2*) for species in the genus *Atriplex* and the Australian Camphorosmeae, along with appropriate outgroups^23^,^24^,^25^,^26^,^54^,^55^,^56^ (Supplementary Data 1 online), and analysed them in a consistent phylogenetic framework. For the *Atriplex* dataset we obtained 90 ITS sequences, which included 33 of the 58 native Australian species^27^. An additional 36 ITS sequences were compiled for the Camphorosmeae dataset which included 33 of the 145 native Australian species^26^. Sequences were aligned using LocARNA^57^ and alignment gaps removed or treated as missing data. Appropriate models of sequence evolution were first determined for each data partition (*ITS1*, 5.8sRNA and *ITS2*) in the *Atriplex* dataset, using Kakusan 4^49^ with the best-fit models being SYM+Γ, SYM+I+Γ and SYM+Γ, respectively. Given the similarity in these models we used the concatenated sequences with the best-fit model for both datasets (*Atriplex* and Camphorosmeae) identified as SYM+Γ (i.e., the GTR+Γ model with equal base frequencies).

Both datasets were analysed in MrBayes 3.2.1^58^ to test whether the data conformed to a strict clock model of sequence evolution. Topological constraints were enforced for both datasets, to ensure trees were correctly rooted, and the GTR+Γ model with equal base frequencies applied. The strict clock model was enforced using a uniform prior on branch lengths, and marginal log likelihoods (MLL) calculated using the stepping stone path sampling procedure. For the relaxed clock model we used the independent gamma rates model (IGR), enforced using the clock variation prior with default value for alpha (0.4) and a power function of 0.9. All analyses were run for 10^6^ iterations with the first 10% discarded as burn-in. Resulting MLL’s were compared with the models rejecting a strict molecular clock for both datasets.

We chose not to employ fossil calibrations directly as this would have required the inclusion of more divergent sequences leading to potential problems as outlined above. Instead we used the *Atriplex* dataset to establish an evolutionary rate for chenopod ITS sequences based on the range of previously published estimates for the root age of the genus^24^, which range from 17.83 to 24.8 Ma. Our approach accounts for variability in the published age estimates but is still subject to any errors associated with the application of the fossil calibrations in the earlier studies. The *Atriplex* dataset was initially analysed in BEAST 1.8^50^ using a GTR+Γ model with equal base frequencies, a strict clock model, and a birth-and-death prior. *Atriplex* was constrained to be monophyletic and a strong normal prior used to fix the root age (*Atriplex* + outgroup *Halimione*) to 17.8, 19.7 or 24.8 Ma according to previously published estimates^24^. Analyses were performed as described above with an MCMC comprising 20 million generations to produce a posterior sample of 10,001 for each run. This analysis returned mean clock rates of 0.00316, 0.0029 and 0.0023 s/s/l/MY, respectively, for the three calibrations.

Subsequent analyses of both datasets employed an uncorrelated lognormal relaxed clock model with a broad uniform prior (UCLD mean: 0–0.00632). The prior range covers the full range of rate values determined in the previous analysis and allows for a minimum two-fold variation in the estimated mean rates. Other parameters and procedures were as described above. For both datasets we used FigTree v1.4.0 to map the distribution of node ages and the ITS rate estimated for each branch in the phylogenies (Supplementary Fig. S2-S3 online). Two sequences with anomalous high ITS rates in the Camphorosmeae dataset (*Malococerca tricornis* AY489224 and *Maireana eriosphaera* EF613602) were excluded from the final analysis (Supplementary Fig. S4 online).

## Additional Information

**Data availability**: Alignments of the nuclear ITS and mtND2 sequences used for dating the origin and diversification of the Australian chenopods (*Atriplex* and Camphorosmeae), spinifex grasses (*Triodia*) and the ATM complex are available at datadryad doi: 10.5061/dryad.0b43t

**How to cite this article**: Norman, J. A. and Christidis, L. Ecological opportunity and the evolution of habitat preferences in an arid-zone bird: implications for speciation in a climate-modified landscape. *Sci. Rep.* **6**, 19613; doi: 10.1038/srep19613 (2016).

## Supplementary Material

Supplementary Information

Supplementary Dataset 1
