# Supplementary material for: Ecological opportunity and the evolution of habitat preferences in an arid-zone bird: implications for speciation in a climate-modified landscape
Source: Sci Rep. 2016 Jan 20;6:19613. doi: 10.1038/srep19613 (PMC4726247; doi:10.1038/srep19613)
Supplement: Supplementary Information [file srep19613-s1.doc]

**Supplementary Information**

Ecological opportunity and the evolution of habitat preferences in an arid-zone bird: implications for speciation in a climate-modified landscape

Janette A Norman and Les Christidis

Supplementary Data 1. Samples used in phylogenetic and demographic analyses of the ATM complex (ND2) and Australian Chenopodiaceae (ITS).

Supplementary Data 2. Preliminary phylogenetic analysis of *Triodia* and the timing of diversification.

Supplementary Figure S1. Branch-specific ND2 rate variation in the ATM complex*.*

Supplementary Figure S2. Branch-specific ITS rate variation in *Atriplex.*

Supplementary Figure S3. Branch-specific ITS rate variation in Camphorosmeae.

Supplementary Figure S4. Branch-specific ITS rate variation in Camphorosmeae with anomalous sequences removed.

Supplementary Table S1 Bayesian analysis of ancestral habitat preferences in the ATM complex.

Supplementary Table S2. Estimated ages for the origin and diversification of Australian Chenopodiaceae.

**Supplementary Data 2 Preliminary phylogenetic analysis of *Triodia* and the timing of diversification.**

The majority of *Amytornis* species are associated with spinifex grasslands (*Triodia*) on rocky escarpments or sandplains^15,16^. Despite the widespread occurrence of spinifex associations a previous study by the authors failed to find support for spinifex grasslands as the MRCA of the ATM complex (0% probability), nor did the study unambiguously identify spinifex as the ancestral habitat of the ATM complex and its sister lineages^19^. In the present study, in which we employed more detailed taxon and habitat partitions, Bayesian model testing also rejected spinifex grasslands as the ancestral habitat of the ATM complex with a low (10%) probability in the unconstrained model. To further test the hypothesis that spinifex grasslands were not the ancestral habitat preference of the ATM complex we surveyed the literature for dated molecular phylogenies of the Poaceae (grasses) that included *Triodia*. *Triodia* (tribe Triodiinae) is a member of the subfamily Chloridoideae that arose 30.9 (24.9 – 36.9) MYA^60^. *Triodia* is identified as a recently evolved endemic Australian lineage and clusters with genera from the Afro-Asian region, a pattern suggestive of a recent colonisation. As dates for the origin and diversification of *Triodia* have not been published we downloaded 47 *Triodia* ITS sequences from GenBank representing 21 named species, along with relevant outgroups (*Aeluropus*, *Orinus* and *Leptochloa*), and analysed them in a Bayesian phylogenetic framework using BEAST 1.8 as outlined in the methods. We employed a mean ITS rate of 0.00413 s/s/l/my with a standard deviation of 0.0005 under a strict clock model and analysed the dataset using a speciation birth-death prior as well as the coalescent constant population size prior. The analyses indicate an age of 6.6 - 6.7 MY for the onset of diversification in *Triodia* (95% HPD 4.6 – 8.8 speciation prior; 4.9 – 9.2 coalescent prior) and an age of 16.2 – 16.7 MY for the initial divergence of *Triodia* from its sister lineage *Aeluropus* (tribe Aeluropodinae) (95% HPD 11.8 – 21.5 speciation prior; 12.0 – 21.5 coalescent prior). The analysis also returned a root age of 22.4 - 23.5 MY for the Cynodonteae which includes *Triodia* plus outgroups (95% HPD 16.8 – 29.5 speciation prior; 17.4 – 31.0 coalescent prior). Given that fossil calibrations have established a mean age for the subfamily Chloridoideae at 30.9 MY (24.9 – 36.9) and the upper end of the 95% HPD for the Cynodonteae overlaps with this, it is plausible that *Triodia* diversified more recently than our current estimate. More detailed analyses using the data from [60] to obtain internal node calibrations would be required to verify this.

Our finding of a recent age (~6.6 MY) for the diversification of *Triodia* is consistent with fossil evidence for a Pliocene origin for grassland ecosystems in arid and semi-arid Australia^13,21^. The combined data supports inferences from our Bayesian trait analysis that *Triodia* is unlikely to be the ancestral habitat of the ATM complex. This would require multiple habitat transitions from an unknown ancestor at ~8.9 MY to *Triodia* at ~6.6 MY, then independent transitions to CS and AES during the Pleistocene. In addition to lacking statistical support from Bayesian model testing (Supplementary Table S2), the complexity of this model seems unlikely given that the earlier origin and diversification of Acacia’s and eucalypts, a habitat currently utilised by the ATM complex, provides a simpler explanation for the evolution of contemporary habitat preferences.

60. Khelladi, Y. B. *et al*. The origin and diversification of C4 grasses and savanna-adapted ungulates. *Glob. Change Biol.* **15**, 2397–2417 (2009).

**Supplementary Figure S1. Branch-specific ND2 rate variation in the ATM complex*.***


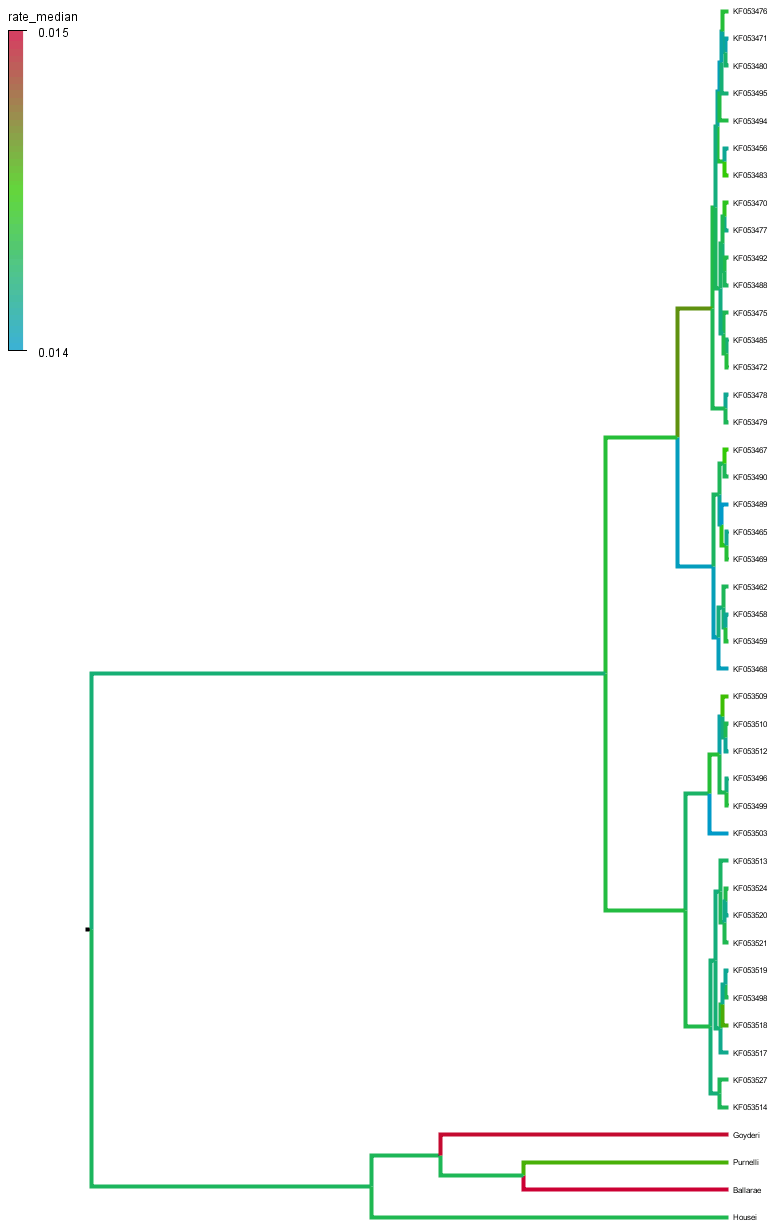


**Supplementary Figure S2. Branch-specific ITS rate variation in *Atriplex*.**

**
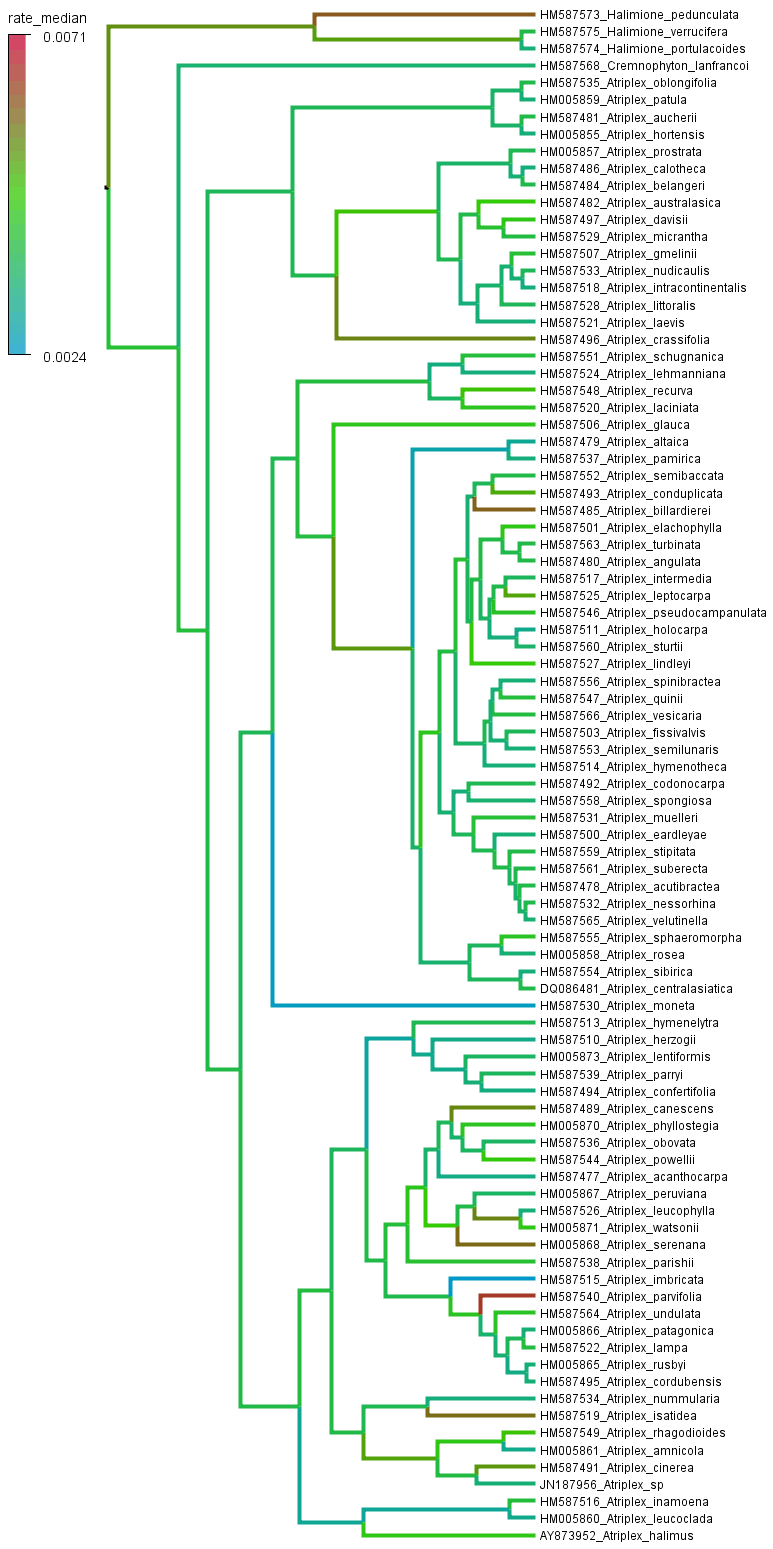
**

**Supplementary Figure S3. Branch-specific ITS rate variation in Camphorosmeae.**


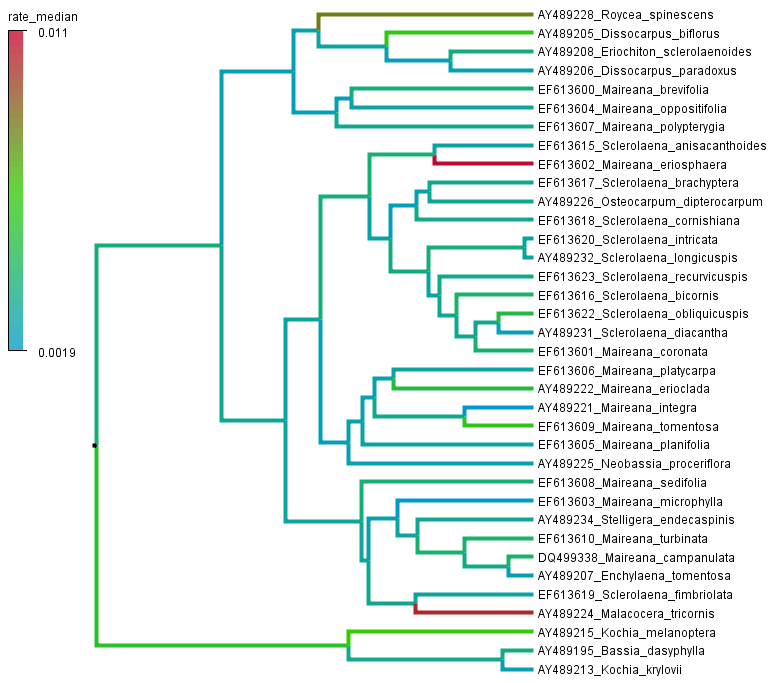


**Supplementary Figure S4. Branch-specific ITS rate variation in Camphorosmeae with anomalous sequences removed.** Scaled to match Figure S1, range 0.0032 – 0.0064.


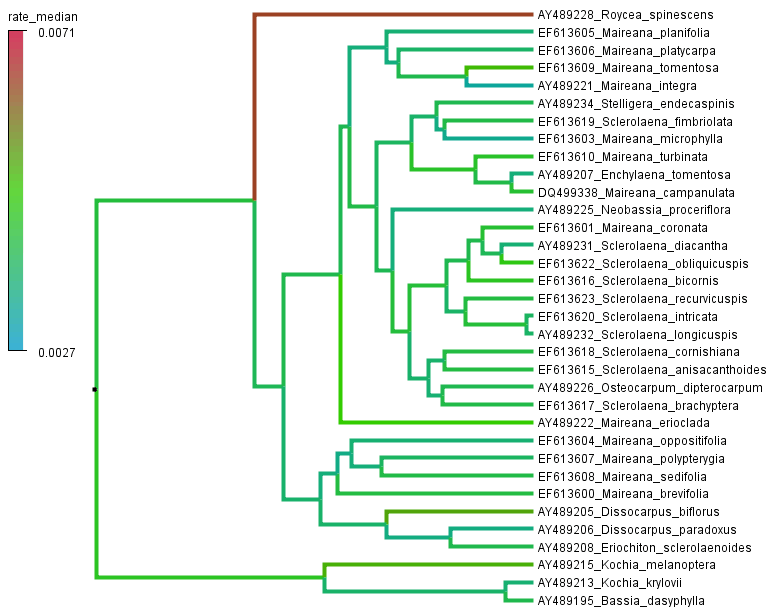


| Node | MRCA | Unconstrained Model  Habitat (% probability)  a b c d | | | | Model A Chenopod ancestor  Habitat (% probability)  a b c d | | | | Model B AES ancestor  Habitat (% probability)  a b c d | | | |
| --- | --- | --- | --- | --- | --- | --- | --- | --- | --- | --- | --- | --- | --- |
| 1 | *modestus + inexpectatus* | 75 | 10 | 7 | 8 | 82 | 7 | 5 | 6 | 68 | 14 | 8 | 10 |
| 2 | *textilis + myall* | 43 | 41 | 7 | 9 | 48 | 40 | 5 | 7 | 34 | 48 | 8 | 10 |
| 3 | *A. textilis + A. modestus* | 57 | 21 | 10 | 12 | 100 | 0 | 0 | 0 | 0 | 100 | 0 | 0 |
| 4 | *A. purnelli + A. ballarae* | 15 | 17 | 50 | 18 | 13 | 15 | 56 | 16 | 18 | 18 | 44 | 20 |
| 5 | Node 4 + *A. goyderi* | 18 | 19 | 36 | 27 | 15 | 18 | 40 | 27 | 20 | 20 | 33 | 27 |
| 6 | Node 5 + *A. housei* | 17 | 19 | 40 | 24 | 15 | 18 | 44 | 23 | 20 | 19 | 36 | 25 |
| Root | Node 3 + Node 6 | 26 | 22 | 29 | 23 | 27 | 21 | 30 | 22 | 23 | 26 | 27 | 24 |
|  | Harmonic Mean | -9.98446 | | | | -10.0696 | | | | -11.8077 | | | |
|  | Bayes Factor | 2.17 | | | | 1.7 | | | | 3.6 | | | |

**Supplementary Table S1 Bayesian analysis of ancestral habitat preferences in the ATM complex.** Values are percent likelihood of each habitat state occurring at that node. Habitat states for each model are (a) chenopod, (b) AES (c) *Triodia* (spinifex grassland) and (d) sandhill canegrass. Model A and model B are described in Figure 2 with Node 3 (shaded) constrained to chenopod and AES, respectively.

| Clade | Origin (Ma) | Crown Age (Ma) | Gene Region | Source |
| --- | --- | --- | --- | --- |
| *Atriplex* | 19.69 |  | *rbcL* | 28 |
|  | 17.83 |  | *atpB-rbcL* | 28 |
|  | 24.8 |  | ITS | 28 |
| Australian *Atriplex* Clade 1 | 9.83 | 7.83 | ITS | 28 |
| Australian *Atriplex* Clade 2 | 6.25 | 4.79 | ITS | 28 |
| Australian Camphorosmeae | 16.4 | 7.5 | ETS | 30 |
|  | 14.75 | 10.35 | *rbcL** | 29 |
|  | 10.3 | 6.3 | *rbcL*^#^ | 29 |
|  | 14.2 | 3.7 | *ndhF** | 29 |
|  | 15.3 | 3.9 | *ndhF*^#^ | 29 |
|  | 14.75 | 2.3 | *atpB*-*rbcL** | 29 |
|  | 16.35 | 5.3 | *atpB*-*rbcL*^#^ | 29 |
|  | 4.7 |  | *rbcL* | 27 |
|  | 5.9 |  | ITS | 27 |

**Supplementary Table S2 Published mean age estimates for the origin and diversification of *Atriplex* and Camphorosmeae.** *, age estimates derived from analyses using the program r8s; #, age estimates derived from analyses using the program BEAST v1.4.8.
